# Retrospective evaluation of high-dose-rate brachytherapy multicriteria planning using physical dose versus radiobiological criteria for prostate cancer

**DOI:** 10.1038/s41598-025-32494-w

**Published:** 2026-01-19

**Authors:** Charles Iorio-Duval, Cédric Bélanger, Éric Vigneault, Luc Beaulieu

**Affiliations:** 1 Département de Physique, de génie physique et d’optique et Centre de recherche sur le cancer, Université Laval, Québec, Québec Canada; 2 Service de Physique Médicale et de Radioprotection, Centre Intégré de Cancérologie, CHU de Québec – Université Laval, Québec, Canada; 3 Département de Physique, Université de Sherbrooke, Sherbrooke, Canada; 4 Axe Oncologie, Centre de recherche CHU de Québec - Université Laval, CHU de Québec, Québec, Canada

**Keywords:** High-dose rate brachytherapy, Radiobiology, TCP, NTCP, UTCP, Prostate Cancer, Computational models, Radiotherapy, Physics

## Abstract

Radiobiological indices can provide insights into treatment efficacy beyond traditional physical dose metrics and potentially facilitate the comparison between various radiotherapy plans. This study investigates the integration of radiobiological indices with standard physical dose criteria to improve high-dose-rate (HDR) brachytherapy plan evaluation and selection process for the treatment of prostate cancers in a multicriteria optimization (MCO) framework. This is accomplished within the framework of a graphics processing unit-based multicriteria optimization algorithm, gMCO. 2000 Pareto-optimal plans for 200 patients were optimized for a 15 Gy HDR brachytherapy boost after external beam radiation therapy (44 Gy in 22 fractions). Tumour control probability (TCP), normal tissue complication probability (NTCP), and uncomplicated tumour control probability (UTCP) were calculated for each plan. Maximizing UTCP alone resulted in insufficient target coverage (target $V_{100} < 90\%$) according to clinical guidelines. Conversely, maximizing target coverage while meeting institutional criteria compromised UTCP significantly (reduction of about 0.09). Selecting plans that met all institutional criteria first, then maximizing UTCP, achieved a balanced compromise between tumour control and normal tissue safety. While combining UTCP and standard dose metrics based on dose-volume histogram (i.e., absorbed dose or physical dose constraints) with MCO can enhance brachytherapy plan optimization, exclusive reliance on standard TCP and NTCP models, using recommended parameters, yields clinically unacceptable plans.

**Supplementary Information:**

The online version contains supplementary material available at 10.1038/s41598-025-32494-w.

## Introduction

Institutional protocols used for radiotherapy treatment planning have the main objective of killing tumourous tissues while preserving as much normal tissue as possible. Those protocols are based on dosimetric criteria meant to quantify the amount of energy absorbed by tissues (i.e., absorbed dose or physical dose). However, the effect of those treatments on patients is affected not only by absorbed dose but also by the context surrounding the treatment (e.g., treatment time, number of fractions, dose-rate, etc.). Two treatments issuing the same physical dose to the patient will have a different impact depending on how the dose is delivered. In that regard, radiobiological indices could be used as optimization criteria to improve current radiotherapy planning algorithms and open new perspectives for decision-making in the clinic. Radiobiological indices consider important radiobiological responses such as reparation and repopulation of cancerous and normal tissue in the treatment efficacy as well as its fractionation effect^1–3^. If well modelled, radiobiological indices could be used as a measurement of the compromise between tumour control and adverse effects to organs at risk (OARs)^4^.

Throughout the years, many radiobiological models have been developed using various statistical techniques^5,6^. Biological effective dose (BED)-based models, generally derived from the linear quadratic (LQ) formalism, were developed to be used as comparative tools between different treatments^3,7^. BED can also be used alone to compare multiple treatments by transforming their physical dose into an equivalence for a specific treatment such as external beam radiotherapy (EBRT) delivered in two Gy fractions (EQD2)^8^. Some models have further included tumour and tissue reparation and repopulation as new assets, that were not considered when considering physical dose only, to better evaluate treatment efficiency^2,9,10^. Tumour control probability (TCP) and normal tissue complication probability (NTCP) models were developed with respect to BED and LQ formalism to quantify the outcome of a treatment based on population-based parameters^11–13^.

This study evaluates the possibility of integrating radiobiological models in the evaluation of high-dose-rate (HDR) brachytherapy treatment plans for prostate cancer. More specifically, this study offers a global radiobiological evaluation with the implementation of a TCP model for target and NTCP models for urethra, bladder, and rectum, which are the standard OARs in brachytherapy treatments for prostate cancer. Thus, the present study proposes a primitive pipeline in phase with AAPM task group 267^6^ to implement a complete radiobiological model for the study of prostate cancer. Furthermore, these models are integrated into the workflow of multicriteria optimization (MCO) to improve plan evaluation and selection based on uncomplicated tumour control probability (UTCP), which combines TCP and NTCP values into a unified evaluation framework. In that regard, recent efficient MCO algorithms were proposed and showed that their integration in clinical workflow could improve both planning efficiency and plan quality^14–21^. However, none of those algorithms characterized the impact of radiobiological models in MCO.

## Methods

All the methods used in the study were approved and carried out in accordance with CHU de Qu$\acute{\textrm{e}}$bec - Universit$\acute{\textrm{e}}$ Ĺaval institutional ethical guidelines and regulations (internal project #: 2022-6140). The research ethics committee further ruled that, given the nature of this study, individual patient consent was not required.

The treatment consists of 44Gy external beam radiotherapy (EBRT) treatment delivered in 2Gy fraction combined with a 15Gy HDR brachytherapy boost (iridium-192) for the treatment of prostate cancer. This treatment is standard in CHU de Québec - Université Laval (CHUQ-UL) for patients with intermediate to high-risk tumour based on their prostate-specific antigen level^22^.

### Experimental data sets

The cohort of patients consisted of 200 prostate cancer cases that were previously treated in CHUQ-UL with an iridium-192 15 Gy HDR brachytherapy boost to EBRT. The planning of the original clinical plans was conducted using Oncentra Prostate v4.2.2 (Elekta, Veenendaal, The Netherlands). The catheters were manually inserted by physicians under transrectal ultrasound (US) guidance. The target, bladder, rectum, and urethra structures were delineated from US images with a slice thickness of 0.5mm. The clinical plans were generated using inverse planning simulated annealing (IPSA)^23^, a well-established optimization algorithm used in brachytherapy planning, followed by manual tweaking of the dwell times as needed. Organ volumes, number of catheters, and number of dwell positions (dwell step of 3 mm) are described in Table 1.

**Table 1:** Characteristics of the cohort of patients used.

	Median	Min	Max
Prostate volume (cc)	43.96	18.18	106.77
Bladder volume (cc)	21.85	1.28	74.34
Rectum volume (cc)	14.68	2.73	35.31
Urethra volume (cc)	1.96	0.86	2.89
Number of catheters	17	14	20
Number of dwell positions	200	128	341

### gMCO algorithm

The MCO algorithm used in this study was a quasi-Newton optimizer designed for parallel plan optimization on GPU architecture (gMCO). gMCO can generate thousands of Pareto-optimal treatment plans within seconds to approximate the Pareto-surface^14,17,20^. In gMCO, piece-wise quadratic functions are used to formulate the objective function^14^. The weighted sum method is used to generate thousands of Pareto-optimal plans with various trade-offs around a population-based *class solution* used as a starting point (see Table S1)^14,20^.

In this study, 2000 Pareto-optimal plans were optimized each time gMCO was executed; this plan number is large enough to approximate the whole solution space given a prescription dose^14^. The 2000 plans were randomly distributed within the solution space using random weights in the objective function *F* as described in Eq. (1)^14^

1 $$\begin{aligned} F = \sum _{s=1}^{N_s} w_s f_s({\textbf {t}}) \end{aligned}$$

where $N_s$ is the number of structures, $w_s$ are the weights, $f_s$ are the individual objective functions (one individual objective function per structure; see Table S1), and ${{\textbf {t}}}$ is the vector of dwell times. As such, by minimizing *F* with different random weight vectors, the Pareto-surface was approximated. The air kerma strength of the source at the time of treatment was used for each patient (median of 24749.81U; range: 15260U to 46480U).

gMCO code is written in C++/CUDA^14^, compiled using Visual Studio Community 2022 (v17.4.5) and CUDA toolkit v12.1. All calculations were executed using an AMD Ryzen 9 5950X 16-Core processor (3.4 GHz and 128GB of RAM) and an NVIDIA GeForce RTX 3090 GPU (24GB of GDDR6X memory and 10 496 CUDA cores).

### TCP model

To estimate tumour control, a Poisson TCP model (Eq. 2) was used^5,24,25^. Both the BED resulting from the EBRT treatment (assuming a constant uniform distribution of prescription dose; such that no dose registration was performed) and the BED resulting from the brachytherapy boost (optimized using MCO) were considered^6^. They were calculated separately and added for each voxel: $BED=BED_\text {EBRT}+BED_\text {Boost}$.

2 $$\begin{aligned} \begin{aligned} TCP=\prod _i \exp \left( -N_0 \frac{v_i}{V_T} \exp (-\alpha \text {BED}_i) \right) \,, \end{aligned} \end{aligned}$$

where $v_i$ is the voxel volume and $V_T$ is the target volume. $\alpha$ is a parameter describing lethal damage of ’single hit’ events^8^. $N_0$ is the number of clonogenic cells in the target (assumed to be uniformly distributed in the target). BED$_i$ is the biological effective dose of the *i*th voxel calculated following Eq. (3)^2^

3 $$\begin{aligned} \begin{aligned} \text {BED}_i= Nd_i\left( 1+ \frac{g}{\alpha /\beta }d_i\right) -\frac{\ln {2}}{\alpha T_p}(T-T_k)\,. \end{aligned} \end{aligned}$$

In Eq. (3), $\beta$ is a parameter describing the lethal damage of ’multiple hit’ events, and $\alpha /\beta$ describes the radiosensitivity of cells^8^. $d_i$ is the physical dose per fraction of the *i*th voxel, and *N* is the number of fractions. $T_p$ is the doubling time of the tumour and $T_k$ is the latent time of cell repopulation (which is considered to be lower or equal to *T*). It is assumed that the EBRT treatment is delivered with 5 fractions a week (22 fractions in total), and the brachytherapy boost is delivered within 3 weeks after EBRT in one fraction^22^. Therefore, the total treatment time *T* in Eq. (3) is considered 49 days for all patients. *g* is the Lea-Catcheside dose protraction factor, which expresses the reparation of the tissue during treatment^2^, the latter which can take about 15-20 minutes for a 15 Gy prescription dose (Eq. 4 with *t* the brachytherapy treatment time)^2,10^.

4 $$\begin{aligned} \begin{aligned} g=\frac{2}{(\mu t)^2}(e^{-\mu t}+\mu t -1)\,, \end{aligned} \end{aligned}$$

In Eq. (4), $\mu$ is the repair rate; $\mu =\frac{\ln {2}}{\tau }$, where $\tau$ is the time to repair half the damage to the tumour. Values for each parameter used in the TCP model are taken from the AAPM task group 137 (TG-137) and in agreement with AAPM task group 267 (TG-267), for prostate tumour cells: $\alpha =0.15$ Gy^-1^, $\beta =0.05$ Gy^-2^, $\alpha /\beta = 3.0$ Gy, $T_p = 42$ days, $T_k = 0$ days, $\tau = 0.27$ h, and $N_0 = 10^6$^6,26^ . In short, the BED formulation in Eq. (3) considers both repopulation and reparation of cancer cells.

### NTCP model

For NTCP calculations, the Lyman-Kutcher-Burman NTCP (LKB) model was used^27–29^. The LKB model follows a sigmoid function and is given by Eq. (5)

5 $$\begin{aligned} \begin{aligned} NTCP=\frac{1}{\sqrt{2\pi }} \int _{-\infty }^k e^{\frac{-x^2}{2}} dx\,, \end{aligned} \end{aligned}$$

with *k* being defined in Eq. (6)

6 $$\begin{aligned} \begin{aligned} k=\frac{\text {gEUD}_{2\text {Gy}}-D_{50}}{mD_{50}}\,. \end{aligned} \end{aligned}$$

In Eq. (6), m is the slope of the dose response curve. The $D_{50}$ in Eq. (6) is the EQD2 dose at which 50% of the patients will encounter the side effect of interest. The generalized equivalent uniform dose (gEUD) is a radiobiological dose weighted on the volume of the organ given by Eq. (7)^29^

7 $$\begin{aligned} \begin{aligned} \text {gEUD}_\text {2Gy}=\left( \sum _i (\text {EQD2})_i^{1/n} \frac{v_i}{V_\text {tot}} \right) ^n\,, \end{aligned} \end{aligned}$$

where *n* is a volumetric dependent parameter, $v_i$ is the voxel volume of the *i*th dose point and $V_\text {tot}$ is the total volume of the organ. EQD2$_i$ is the biological effective dose delivered in 2 Gy fraction of the *i*th voxel and can be expressed as in Eq. (8)

8 $$\begin{aligned} \begin{aligned} \text {EQD2}_i=Nd_i\left( \frac{1+\frac{d_i}{\alpha /\beta }}{1+\frac{2}{\alpha / \beta }}\right) =\frac{\text {BED}_i}{1+\frac{2}{\alpha / \beta }}=\frac{\text {BED}_{(EBRT)i}+\text {BED}_{(Boost)i}}{1+\frac{2}{\alpha / \beta }}\,. \end{aligned} \end{aligned}$$

Note that the BED for OARs did not include normal tissue repair and repopulation. The NTCP gives the probability that certain side effects occur on the basis of previously recorded data. In this paper, three different side effects were considered: urethral stricture, rectum severe proctitis, necrosis, stenosis and fistula, and bladder contracture and severe volume loss^11,30,31^ (see Table 2).

**Table 2:** Radiobiological parameters used in the LKB model for the calculation of NTCP.

Side effect	Urethra stricture	Rectum proctitis, necrosis stenosis and fistula	Bladder contracture and severe volume loss
m	0.23 $^{b}$	0.15$^{a}$	0.11$^{a}$
D50 (Gy)	116.7 $^{b}$	80$^{a}$	80$^{a}$
n	0.3 $^{b}$	0.12 $^{a}$	0.5 $^{a}$
$\alpha /\beta$ (Gy)	5 $^{b}$	5.4 $^{c}$	7.5 $^{c}$

$$^{a}$$^11^ $$^{b}$$^30^ $$^{c}$$^31^

Another useful radiobiological index is the probability of injury ($P_I$)^32^. $P_I$ combines the NTCP of all OARs in a single value as defined in Eq. (9)^32^

9 $$\begin{aligned} P_I = 1 - \prod _j^{N_{OARs}}(1 - NTCP_j)\,, \end{aligned}$$

where $N_{OARs}$ is the number of OARs, and NTCP$_j$ is the NTCP of the *i*th organ. The ideal value of $P_I$ is 0^32^.

### UTCP model

The UTCP is a quantitative indicator of the trade-off between TCP and NTCPs defined in Eq. (10)^33^

10 $$\begin{aligned} UTCP=TCP \cdot \prod _j^{N_{OARs}}(1 - NTCP_j)\,, \end{aligned}$$

A UTCP value of 1 means that both tumour control and OARs sparing are perfectly met (ideal treatment outcome). In this study, all OAR NTCPs are considered to have the same weight for means of simplicity. However, in subsequent studies, different OAR NTCPs could have different weights in the UTCP equation depending on their rate of occurrence or on what side effect the clinician wants to prioritize. UTCP provides a combined measure that reflects both tumour control and normal tissue sparing, facilitating the ranking of the plans within a unified evaluation framework.

### Impact of the prescription dose

The HDR brachytherapy boost prescription (single fraction) was used to set the gMCO algorithm (see Table 3) and is in theory the dose that would be administered to the whole tumour. In this study, it was varied from 1 Gy to 20 Gy to measure its impact on radiobiological indices. This was done by rescaling the dose parameters ($D_\text {min}$ and $D_\text {max}$) in the gMCO class solution in Table S1 for each structure according to the target prescription. For each prescription, 2000 Pareto-optimal plans were generated with gMCO. Thus, this gave 2000 plans/fraction $\times$ 20 fractions/patient = 40 000 plans/patient, and a total of 200 patients $\times$ 40,000 plans/patient = 8,000,000 optimized plans. This experiment is referred to as TCP_TG267_.

### Impact of radiobiological parameters

#### TCP parameters

The experiment in Section “Impact of the prescription dose” was repeated by recalculating the TCP values of all gMCO-generated plans with a range of previously proposed radiobiological parameters (^26,34,35^) to estimate their impact on radiobiological results (see Table S2 and Fig. S1). The $\alpha$ parameter was probed (see experiments c and d) to observe its influence on TCP and UTCP while maintaining a constant $\alpha / \beta$. The impact of varying $\alpha$ was further evaluated for fixed $\beta$ values in the case of a 15 Gy prescription boost (see Figs. S2 to S5).

#### NTCP parameters

The impact of $\alpha /\beta$ was also estimated for the NTCP models. The experiment in Section “Impact of the prescription dose” was repeated by recalculating the NTCP values for all OARs at the same time for $\alpha /\beta$ values ranging between 1 and 10 Gy while keeping the parameters constant for the TCP model (TCP_TG267_).

### Plan selection scenarios and dosimetric criteria

To test different plan selection scenarios based on radiobiological metrics and dosimetric criteria (physical dose), gMCO-generated plans with a 15 Gy prescription dose from Section “Impact of the prescription dose” were considered (i.e., a subset of 2000 plans/patient $\times$ 200 patients = 40,000 plans was used). Two sets of dosimetric criteria (i.e., clinical goal constraints) based on physical dose for plan evaluation and plan selection were used (see Table 3)^17,20^. Those criteria are currently used as guidelines at CHUQ-UL for a 15 Gy HDR brachytherapy boost (single fraction) to EBRT. In Table 3, institutional plus (INST+) criteria defined the planning aims (i.e., more stringent constraints), while institutional (INST) criteria defined the baseline criteria (i.e., less stringent constraints and minimum requirements for treatment plan acceptability).

**Table 3:** Dosimetric criteria used for a 15 Gy HDR brachytherapy boost delivered in a single fraction.

DVH indice	INST	INST+
Target $V_{100}$ (%)	$\ge 90$	$\ge 95$
Target $V_{150}$ (%)	$< 45$	$< 40$
Target $V_{200}$ (%)	$< 20$	$<16$
Bladder $V_{75}$ (cc)	$<2$	$<1$
Rectum $V_{75}$ (cc)	$<2$	$<1$
Rectum $V_{100}$ (cc)	$=0$	$=0$
Urethra $D_{10}$ (Gy)	$<18$	$<17.7$
Urethra $V_{125}$ (cc)	$=0$	$=0$

**Fig. 1:**
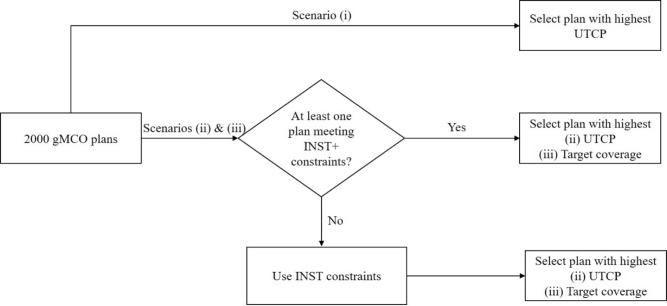
Plan selection scenarios.

Three simple plan selection scenarios were tested and compared for each patient (see Fig. 1):

(i) In the first scenario, out of the 2000 gMCO Pareto-optimal plans, the plan that maximized UTCP directly (unconstrained solution space) was selected;
(ii) In the second scenario, the plan that maximized the UTCP while meeting physical dose criteria in Table 3 was selected. In other words, INST+ constraints were prioritized first; if INST+ constraints cannot be met simultaneously for at least one plan (out of 2000 plans), INST constraints were used instead;
(iii) In the third scenario, the plan that maximized the target coverage following the same priority as in scenario (ii) was selected.

The results of the plan selection scenarios was compared with the original clinical plans (approved by physicians).

## Results

### Impact of the prescription dose

**Fig. 2:**
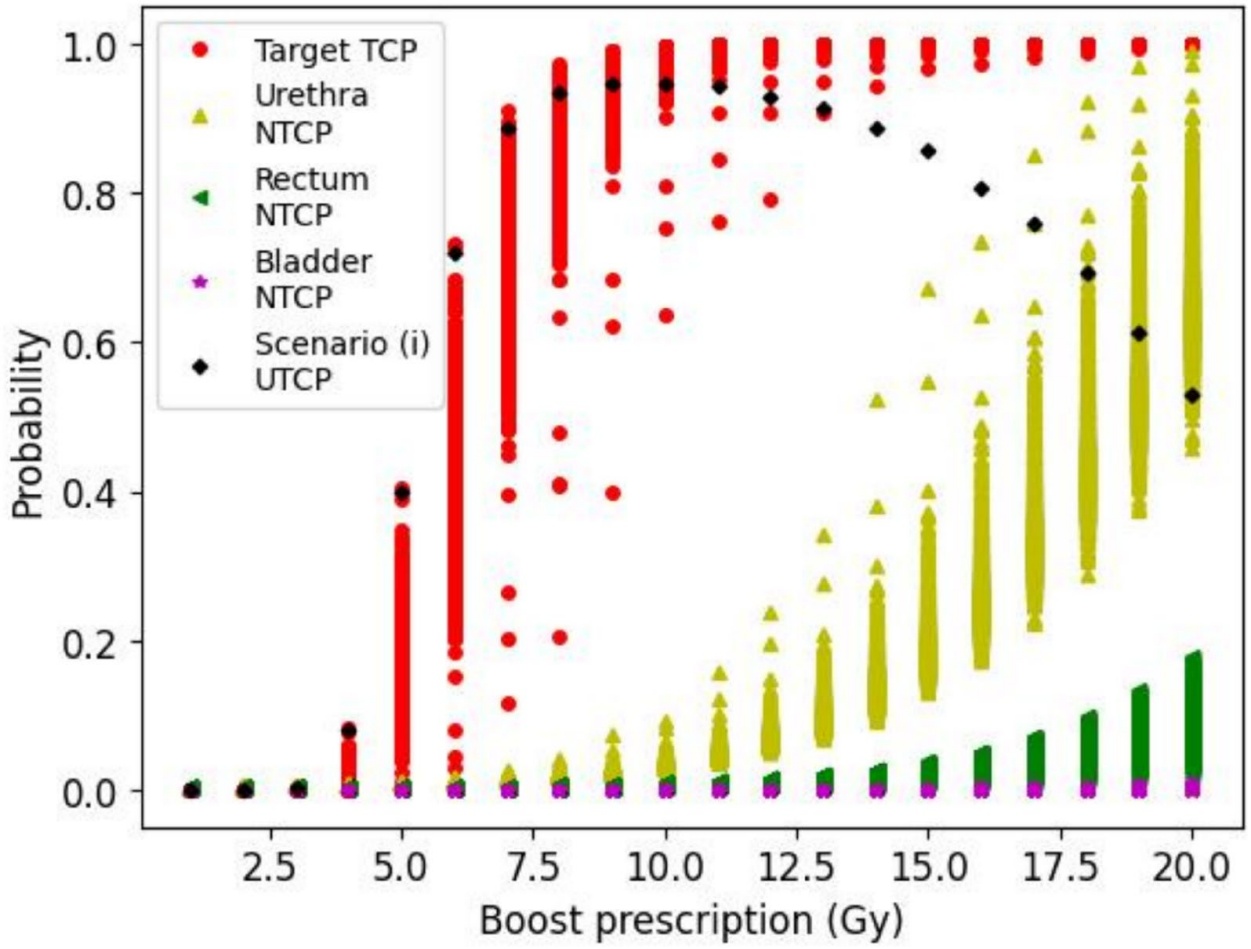
Effect of the prescription dose of the HDR brachytherapy boost treatment (single fraction) to EBRT on the TCP and NTCPs for one random example case. The radiobiological indices of the 2000 gMCO Pareto-optimal plans (for each prescription) prior to plan selection and UTCP value of the plan selected under scenario (i) are depicted.

The variation of different radiobiological indices of 2000 gMCO Pareto-optimal plans with regards to the prescription prior to plan selection under scenarios (i)–(iii) is shown in Fig. 2 for one example case. As expected, radiobiological parameters evolved in a sigmoid manner when increasing the dose prescription with a gap between the TCP and NTCPs. The black diamond marker illustrates the selected plan under scenario (i), where a maximum UTCP of 0.95 was obtained around 10 Gy.

**Fig. 3:**
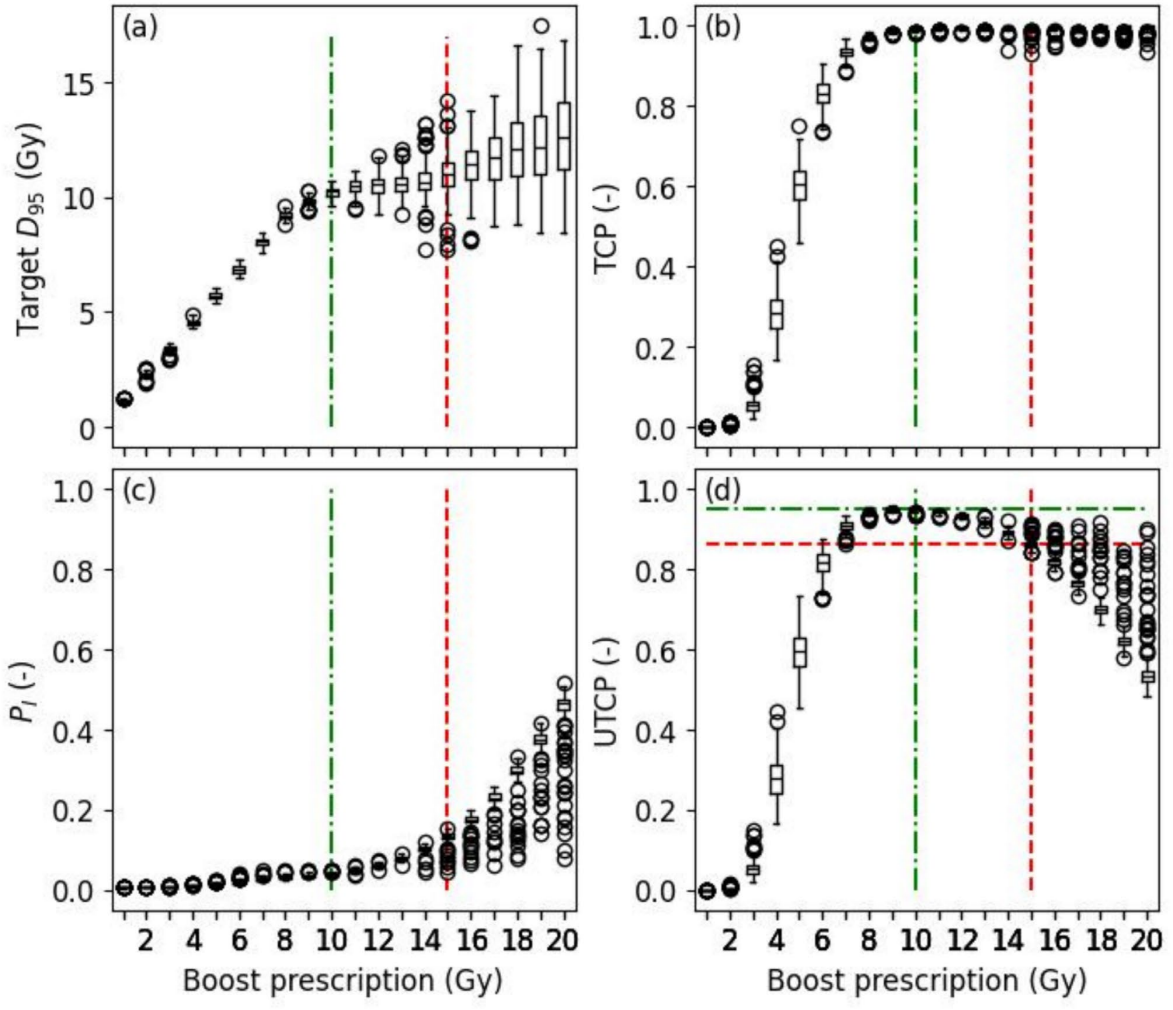
Effect of the prescription dose of the HDR brachytherapy boost treatment (single fraction) to EBRT on target $D_{95}$, TCP, $P_I$, and UTCP. Values are obtained by plan selection under scenario (i) for each prescription dose over the whole cohort of patients. The red dashed lines illustrate the median UTCP according to the current prescription used at CHUQ-UL (15 Gy). The green dashed-dotted lines illustrate the prescription (10 Gy) that maximizes the median UTCP for the whole cohort of patients.

In Fig. 3, the boxplot distribution of selected plan (via scenario [i]) for different HDR brachytherapy boost prescription doses across the whole cohort of patients is shown. With a low brachytherapy prescription dose ($\le 4$ Gy in Fig. 3d), the UTCP was below 0.5. This was because the TCP was also low (i.e., $< 0.5$ in Fig. 3b) for those prescription doses, while $P_I$ was close to 0.

Figure 3d further showed that the UTCP was maximized with a prescription dose of 10 Gy (median value of 0.95). Above 10 Gy, the median UTCP decreased while the boost prescription dose increased. This was because the probability of injury to OARs ($P_I$ in Fig. 3c) increased with the prescription dose, while the TCP was already maximized at 10 Gy. Furthermore, the median $D_{95}$ increased linearly up to 10 Gy; above 10 Gy, plans with favorable trade-offs for OARs sparing and lower target dose were selected to maximize the UTCP.

### Impact of radiobiological parameters

#### TCP parameters

The impact of the radiobiological parameters of the TCP model on the UTCP was characterized in supplementary material (Fig. S1 and Table S2). Overall, increasing $N_0$ in the TCP Poisson model had the effect of shifting the maximum UTCP value toward higher boost doses (i.e., 11 Gy for $N_0 = 10^7$ and 13 Gy for $N_0 = 10^8$). In addition, because NTCP values for OARs did not change, the highest achievable UTCP value decreased with $N_0$ (i.e., from 0.95 with $N_0 = 10^6$ down to 0.86 with $N_0=10^8$). When decreasing $\alpha$ (while keeping $\alpha /\beta =3$ Gy), a shift toward higher boost dose was also observed in the UTCP (i.e., maximum UTCP of 0.66 at 16 Gy for $\alpha =0.09$ Gy^-1^). When using parameters reported by Brenner et al. ($\alpha = 0.036$ Gy^-1^ and $\alpha /\beta =1.5$ Gy)^34^, the TCP was zero up to 16 Gy, such that the UTCP was below 0.2 for all prescription doses, which did not align with clinical outcome.

The sensitivity of the UTCP model to parameters $\alpha$ and $\alpha /\beta$ for prostate was further described by Figs. S2 to S5 for a 15 Gy brachytherapy boost prescription. In general, a maximum UTCP value (up to 0.86) was reached when increasing the values of $\alpha$ (i.e., $\alpha / \beta$) and with a fixed $\beta$ value. Also, the higher was the value of (fixed) $\beta$, and for the same $\alpha /\beta$ ratio (e.g., 1.5 Gy and 3 Gy), the higher was the UTCP. Furthermore, the UTCP reached its maximum more quickly. According to clinical outcome, reasonable values of $\beta$ for prostate were found to be between 0.03 and 0.05 Gy$^{-2}$ ($\beta =0.024$ Gy$^{-2}$ in Fig. S2 was not realistic).

#### NTCP parameters

The sensitivity of the NTCP models with respect to the $\alpha /\beta$ parameter was presented in Fig. S6. At 15 Gy, the median $P_I$ ranged between 0.06 and 0.58, and UTCP ranged between 0.4 and 0.93. Furthermore, the treatment window (i.e., width of boost prescription dose for a given UTCP value) increased when $\alpha /\beta$ increased (e.g., width of 6 Gy to have UTCP > 0.8 with $\alpha /\beta =1$ Gy, and width of 10 Gy to have UTCP > 0.8 with $\alpha /\beta = 6$ Gy). Overall, with $\alpha /\beta > 4$ for OARs the different models seem pretty similar at a boost of 15 Gy. Increasing the $\alpha /\beta$ ratio for the OARs in the NTCP models increased the UTCP value (up to 0.96 at 10 Gy).

### Plan selection scenarios

**Fig. 4:**
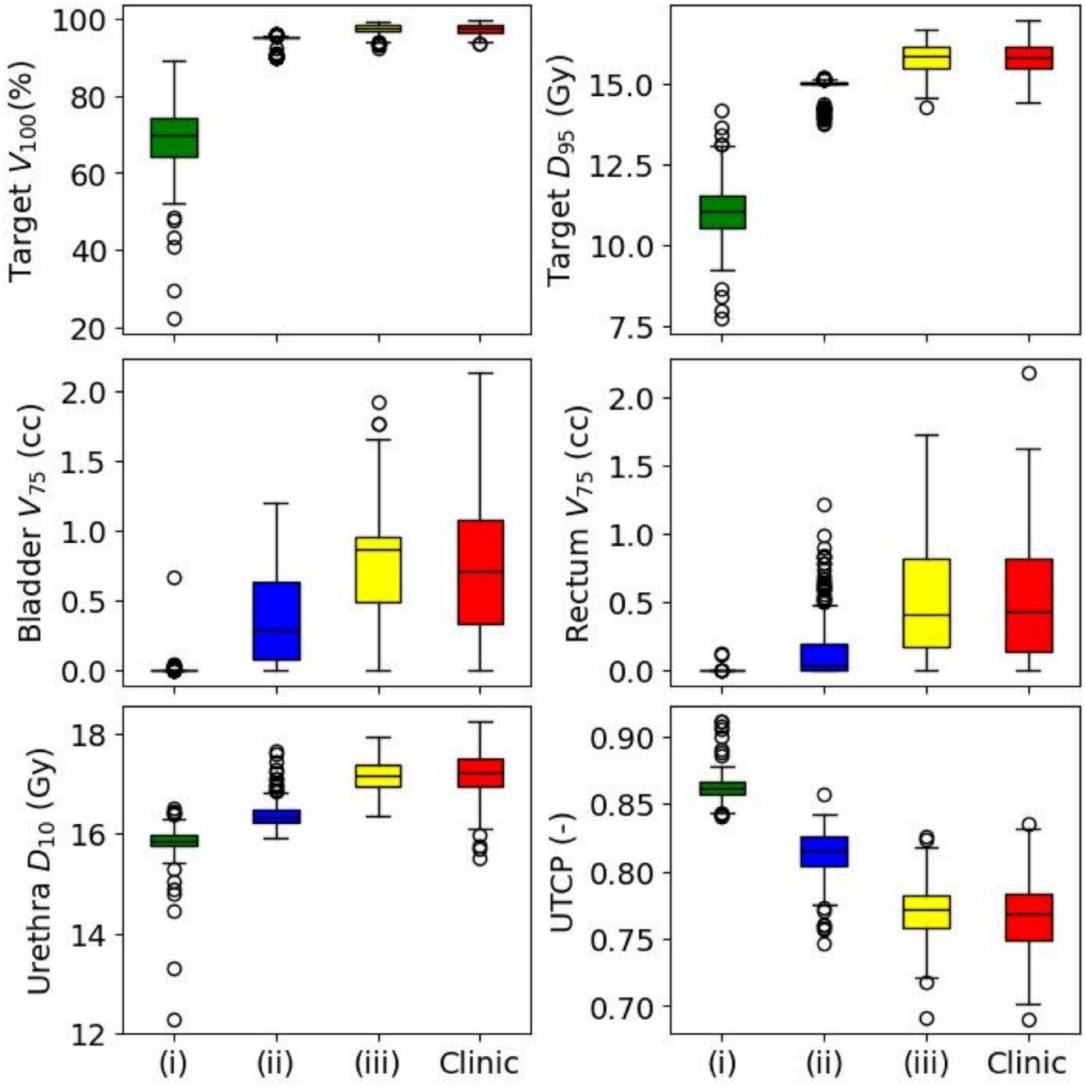
Key indices obtained with three different plan selection scenarios from 2000 gMCO Pareto-optimal plans with 15 Gy prescription (single fraction) boost dose to EBRT. The results of the clinical plans are also depicted.

Figure 4 showed key DVH indices and UTCP obtained from three different plan selection scenarios with gMCO and original clinical plans (see companion Fig. S7 for other radiobiological parameters). In Fig. 4 for scenario (i), the median UTCP reached a value of 0.86. When looking at physical dose indices, scenario (i) favored plans with lower target coverage (median of 70.0% in the target $V_{100}$). On the other hand, OARs were better spared compared with the two other scenarios (e.g., median decrease of 0.5 Gy and 1.3 Gy in the urethra $D_{10}$ compared with scenarios [ii] and [iii], respectively).

For scenario (ii), the UTCP (median of 0.82) was lower with the introduction of physical dose constraints for plan selection (decrease of 0.04 in UTCP compared with scenario [i]). This was because the constraint on the minimum target coverage worsened OARs sparing (NTCP) without gains in the TCP (saturated with a 10 Gy prescription; see Fig. 3). Therefore, gains in the target coverage with a 15 Gy prescription were marginal for the TCP, but detrimental for the UTCP according to the models.

For scenario (iii), the UTCP was the lowest (median value of 0.77), because no significant gains were expected in the TCP when increasing the target coverage for a 15 Gy prescription. Nevertheless, as seen in Fig. 4, the UTCP under scenario (iii) was similar to what is observed in our clinic (median UTCP of 0.77 for clinical plans). This suggested that physicians in our institution prioritized the target coverage over OARs sparing given the current prescription.

## Discussion

According to radiobiological models used with TG-137/TG-267 parameters (Fig. S1), results show that a boost of 10 Gy maximizes the UTCP, and that a boost of 8 Gy would achieve a higher UTCP compared to the currently prescribed dose of 15 Gy in our clinic (recommendations of GEC-ESTRO ACROP^36^), meaning that dose de-escalation would be clinically beneficial according to the currently recommended model parameters. However, this observation differs with clinical outcomes, given that the increase in BED with hypofractionated regimens (HDR boost to EBRT) correlated with an improvement in biochemical control^22,37^. Those conclusions suggest that current radiobiological models and/or model parameters (e.g., see Fig. S1) cannot fully capture the outcome of large doses delivered by HDR brachytherapy treatments. In that regard, there are undergoing debates about whether the LQ model is valid for large dose per fraction (i.e., > 8–10 Gy)^38–40^, which would overestimate the TCP and underestimate the NTCP if the underlying LQ model is invalid. Therefore, this would shift the maximum UTCP towards a higher dose per fraction in Fig. 3 (assuming that the TCP has the greatest impact). Furthermore, Fig. S1 shows that the choice of radiobiological parameters has significant impact on the models and their clinical interpretation, as a wide range of boost prescription dose can maximize the UTCP when changing TCP parameters. Nevertheless, the rationale to introduce UTCP into MCO in brachytherapy is to provide additional information to planners about the ranking of plans with a single value^41^ and hopefully to enhance the plan selection process with new perspectives as MCO gains more ground. While the absolute value of UTCP (i.e., models’ parameters) was not calibrated against real clinical data, relative comparison between plans using UTCP is still meaningful and complementary to standard criteria based on dose metrics. It also paves the way to incorporate radiobiological metrics into clinical practice.

As showed in Fig. 4, selecting plans solely based on radiobiological parameters (i.e., scenario [i]) does not lead to clinically acceptable plans according to clinical guidelines for a single fraction brachytherapy boost of 15 Gy (see Table 3) because of low target coverage ($<90\%$). However, it is possible to improve the UTCP while meeting physical dose constraints (scenario [ii] vs. scenario [iii]). In the clinic, this suggests that radiobiological models (if accurate enough) could convey additional information on plan quality and trade-offs during the plan selection process. Adding the calculation of TCP and NTCP is clinically feasible with gMCO given that the mean time to calculate DVH curves and radiobiological indices for 2000 Pareto-optimal plans is only 0.9 s (range: 0.4–5.6 s). Moreover, the large spread of NTCP for urethra (variation of about 0.6) compared with TCP (variation of about 0.05) with a prescription of 15 Gy (see Fig. 2) suggests that there is room to optimize radiobiological parameters directly. In other words, there are trade-offs that allow lower urethra NTCP while maintaining high TCP with limited impact on bladder and rectum NTCPs.

This study has some limitations. Because patients were treated using US-based planning, bladder and rectum structures were not fully delineated by the physician (i.e., only visible parts closest to the target were contoured); this can have an impact on the NTCP results. Although not characterized in the current study, it is well known that other parameters such as the imaging modality used, slice thickness between images (i.e., contours), and contour variability between observers have impact on volumes and DVH parameters^42,43^. Therefore, in addition to radiobiological parameters uncertainties (see Figs. S1–S6), it is reasonable to expect that those uncertainties propagate throughout TCP and NTCP calculations, such that care should be taken when interpreting radiobiological metrics in an absolute manner. This study assumed a time-averaged uniform dose-rate throughout the treatment such that the results underestimate the TCP compared with time-dependent dose-rate^10^. The EBRT dose was assumed to be uniformly distributed with each organ, such that no dose registration was performed between the EBRT dose and the brachytherapy dose. The impact of radiobiological parameters was characterized for a limited set of values and parameters ($\alpha$ and $\alpha /\beta$ for TCP and $\alpha /\beta$ for NTCP); see Figs. S1 and S6). In that regard, fully exploring a wide range of possible values reported in the literature (i.e., brute force approach) for TCP and NTCP models is computationally expensive (i.e., calculation time and data storage). This stresses the importance of either refining the models, reducing uncertainties in parameters or implementing robust optimization algorithms that can consider uncertainties. In that regard, robust optimization was successfully applied to contour uncertainties in brachytherapy^44^ and is gaining attention in intensity modulated ion therapy for radiobiology^45^. Direct optimization of radiobiological parameters was not implemented, so that the presented TCP, NTCP, and UTCP results are not guaranteed to be optimal. However, gMCO explores a wide solution space, such that clear trends were highlighted when adding radiobiological models to MCO for plan evaluation. Even though radiobiological models used in this study are simplistic and population-based (e.g. assuming fixed values rather than probability distribution of parameters, uniform distribution of tumour cells, uniform distribution of EBRT dose, etc^25^), it is still of crucial importance to characterize their clinical impact as they could become patient-specific in the future with the evolution of biomarkers^4^.

## Conclusion

This study has evaluated radiobiological models to enhance plan evaluation and selection within a multicriteria optimization framework. Currently recommended model parameters lead to discrepancies between predicted dose levels for optimal UTCP and clinical observations for prostate HDR brachytherapy boost. However, when changing parameters in the TCP model, a wide range of optimal boost dose (i.e., maximization of UTCP) was observed. Including radiobiological indices in the plan evaluation and selection in a MCO workflow during plan navigation could help to convey additional information about clinical trade-offs. However, even if those tools are promising and useful, results show that it would not be possible to use them without also considering physical dose constraints, as they give misleading indications on the plan quality i.e. unacceptable target coverage with regards to clinical guidelines. Future work should focus on prospective validation of radiobiological metrics against clinical outcomes to assess their predictive reliability in clinical practice.

## Supplementary Information

Supplementary Information.

## Data Availability

The plans generated and patients’ imaging data used for this study are not publicly available due to institutional policies on sensitive medical data. For any requests regarding the data used this study, please contact Luc Beaulieu (Luc.Beaulieu@phy.ulaval.ca).
