# Supplementary material for: Retrospective evaluation of high-dose-rate brachytherapy multicriteria planning using physical dose versus radiobiological criteria for prostate cancer
Source: Sci Rep. 2026 Jan 19;16:2561. doi: 10.1038/s41598-025-32494-w (PMC12820064; doi:10.1038/s41598-025-32494-w)
Supplement: Supplementary file 1 — Supplementary Information. [file 41598_2025_32494_MOESM1_ESM.pdf]

Supplementary Information to: Retrospective evaluation of high-dose-rate brachytherapy multicriteria planning using physical dose versus radiobiological criteria for prostate cancer

Charles Iorio-Duval[1,2,†], Cédric Bélanger[2,†], Éric Vigneault[2], and Luc Beaulieu[2,3,*]

[1]Département de physique Université de Sherbrooke, Sherbrooke, Québec, Canada
[2]Service de physique médicale et de radioprotection, Centre intégré de cancérologie, CHU de Québec – Université Laval et Centre de recherche du CHU de Québec, Québec, Canada
[3]Département de physique, de génie physique et d'optique et Centre de recherche sur le cancer de l'Université Laval, CHU de Québec, Québec, Canada
[†]These authors contributed equally to this work
[*]E-mail for correspondence: Luc.Beaulieu@phy.ulaval.ca

Table S1: Class solution used to formulate piece-wise quadratic objective functions for 15 Gy prostate boost HDR treatment in gMCO (Surface: surface dose calculation points, Volume: volume dose calculation points). w_{min} and w_{max} are weights associated with minimum (D_{min}) and maximum (D_{max}) doses.

Structure	Surface				Volume			
	w_{min}	D_{min}(Gy)	D_{max}(Gy)	w_{max}	w_{min}	D_{min}(Gy)	D_{max}(Gy)	w_{max}
Target	200	15	-	-	200	15	-	-
Urethra	30	14.25	16.5	140	30	14.25	16.5	140
Bladder	-	-	7.5	32	-	-	7.5	32
Rectum	-	-	7.5	7	-	-	7.5	7
Normal tissue	-	-	-	-	-	-	18.75	15

Table S2: Radiobiological parameters used for the calculation of TCP (companion table of Figure S1)

	α (Gy^{-1})	β (Gy^{-2})	α/β (Gy)	N_0 (-)	T_p (days)	T_k (days)	μ (hours)
$\text{TCP}_{\text{TG267}}$	0.15	0.05	3.0	10^6	42	0	0.27
TCP_{a}	0.15	0.05	3.0	10^7	42	0	0.27
TCP_{b}	0.15	0.05	3.0	10^8	42	0	0.27
TCP_{c}	0.12	0.04	3.0	10^6	42	0	0.27
TCP_{d}	0.09	0.03	3.0	10^6	42	0	0.27
TCP_{e}	0.036	0.024	1.5	10^6	42	0	0.27

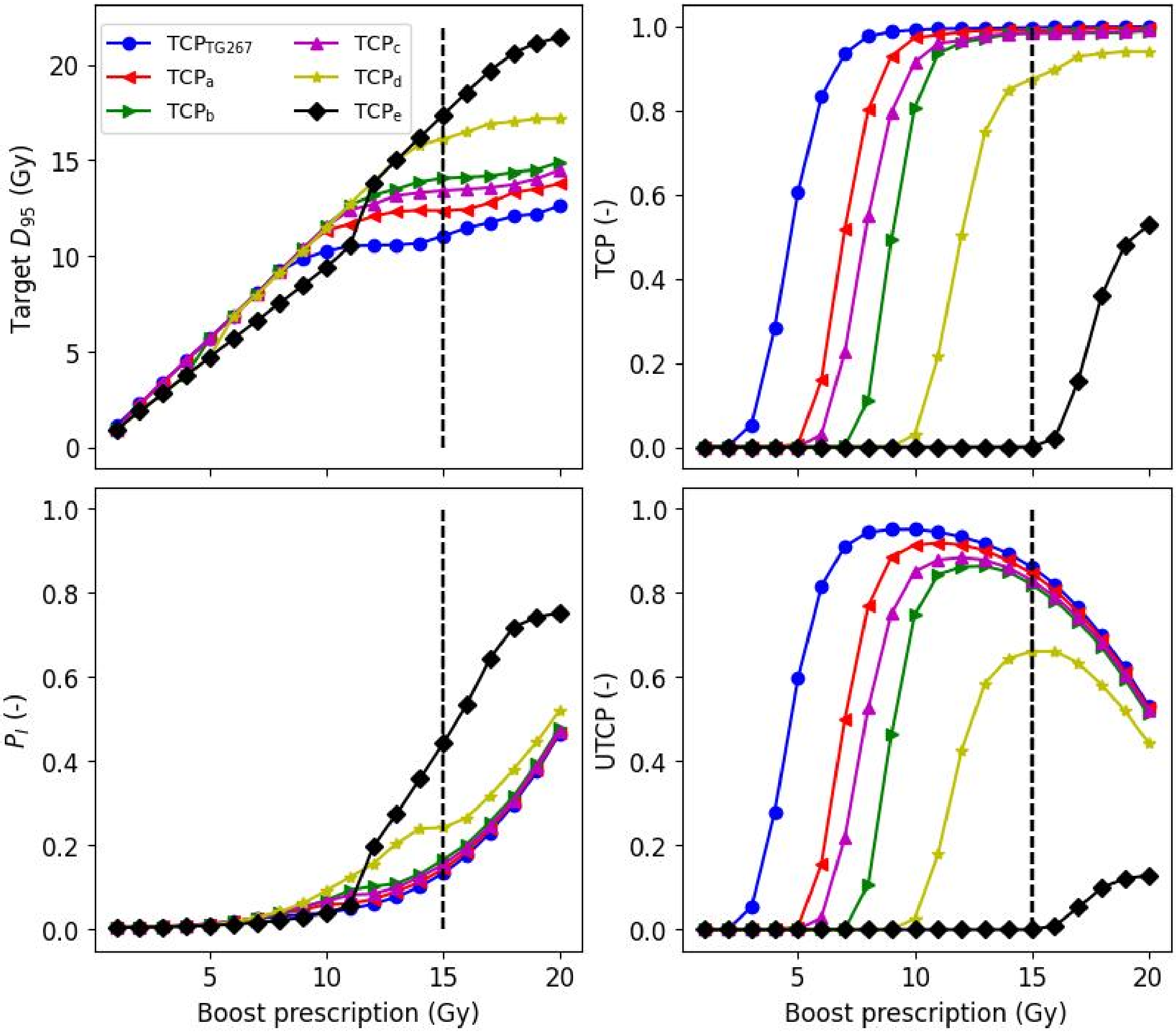

Figure S1: Impact of the radiobiological parameters (TCP model) for different HDR brachytherapy boost prescriptions (delivered in a single fraction) to external beam radiation therapy (EBRT) on the (a) Target D_{95}, (b) TCP, (c) P_I, and (d) UTCP. The curves show median values over the whole cohort of patients when selecting plan with the highest UTCP from the 2000 gMCO Pareto-optimal plans for each prescription. The labels are defined Table S2. The black dashed lines illustrate the current boost prescription dose used at CHUQ-UL center (15 Gy).

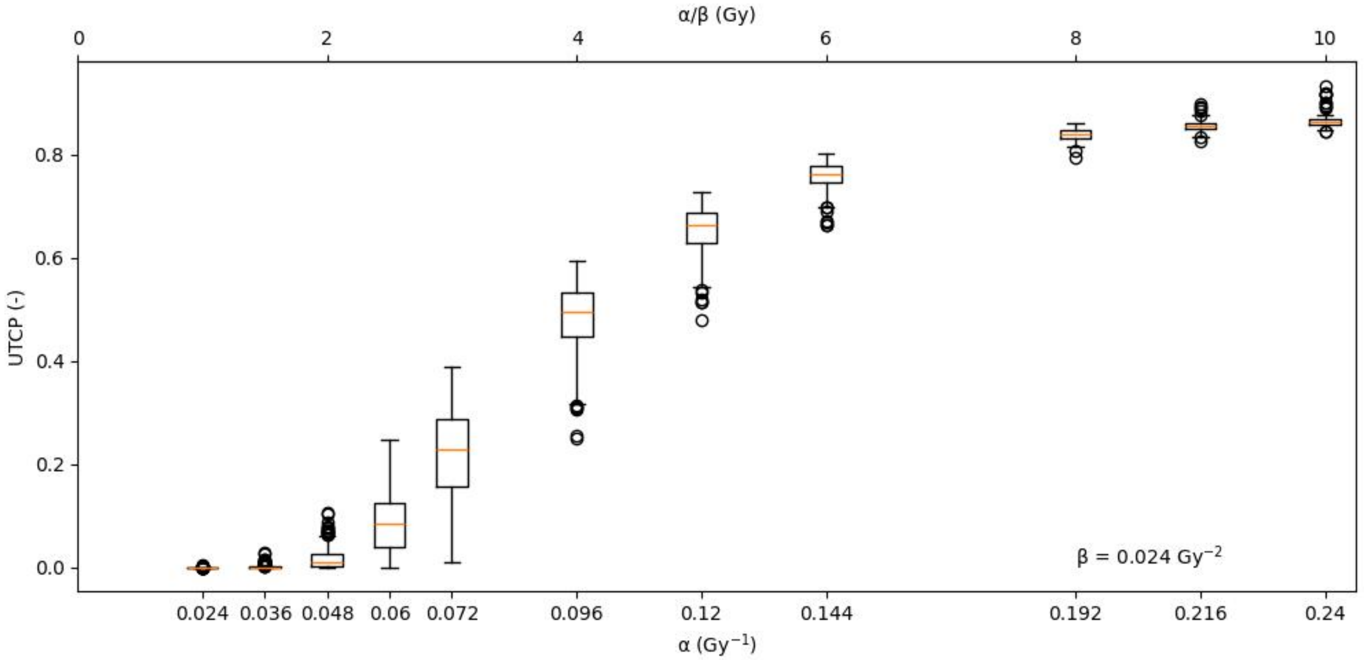

Figure S2: Impact of the α parameter (TCP model) for a 15 Gy HDR brachytherapy boost prescriptions while keeping a constant value of $\beta = 0.024$ Gy^{-2}. The boxplot shows UTCP values over the whole cohort of patients when selecting the plan with the highest UTCP from the 2000 gMCO Pareto-optimal plans for each α value. The NTCP parameters for OARs are kept constant by using values in Table 2.

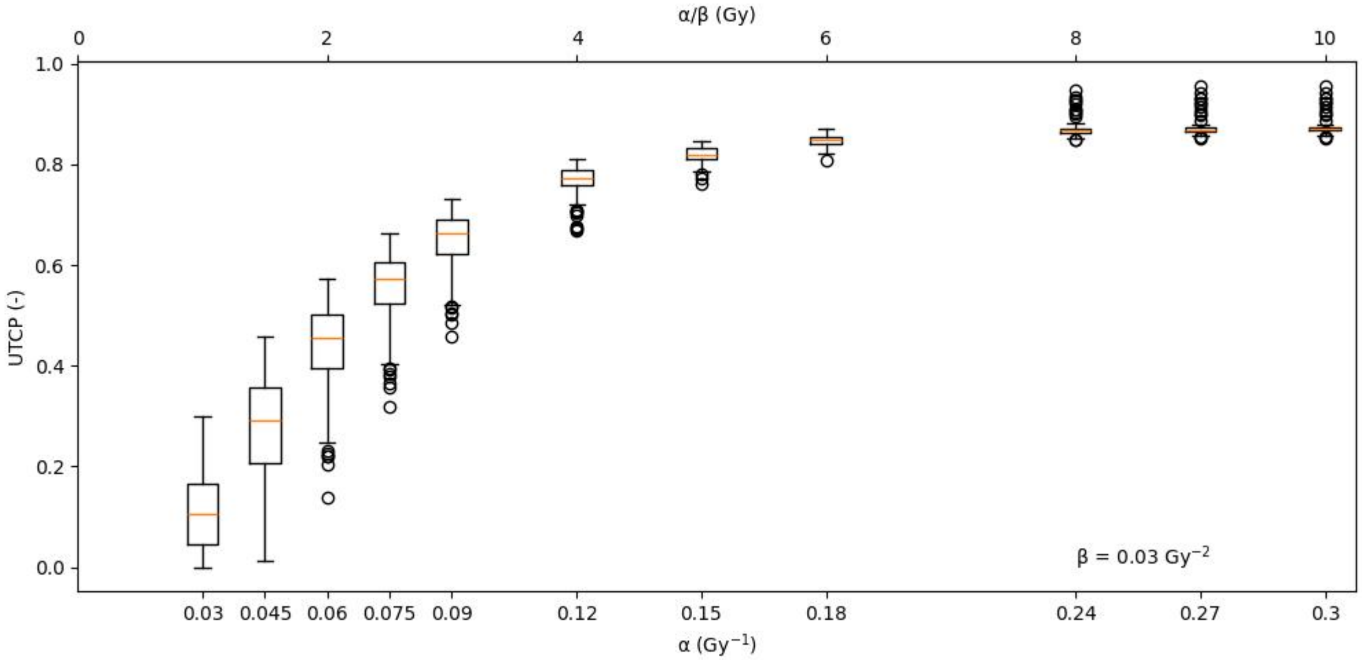

Figure S3: Impact of the α parameter (TCP model) for a 15 Gy HDR brachytherapy boost prescriptions while keeping a constant value of $\beta = 0.03$ Gy^{-2}. The boxplot shows UTCP values over the whole cohort of patients when selecting the plan with the highest UTCP from the 2000 gMCO Pareto-optimal plans for each α value. The NTCP parameters for OARs are kept constant by using values in Table 2.

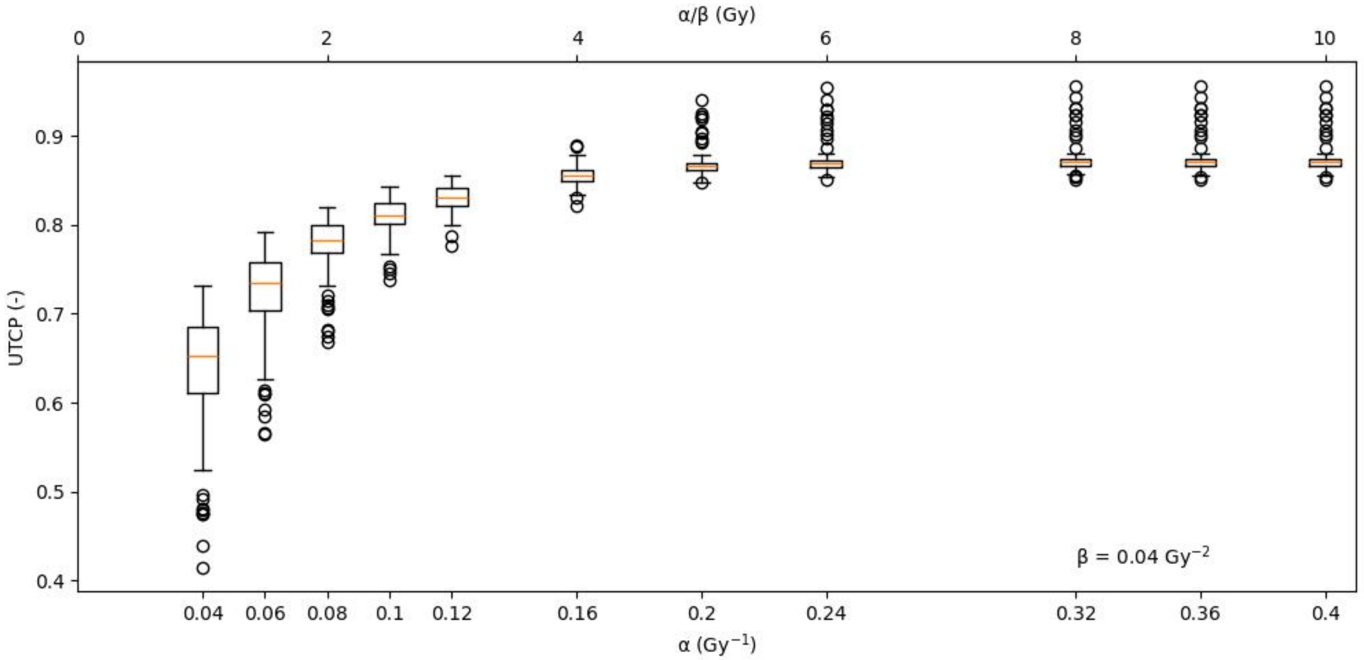

Figure S4: Impact of the α parameter (TCP model) for a 15 Gy HDR brachytherapy boost prescriptions while keeping a constant value of $\beta = 0.04$ Gy^{-2}. The boxplot shows UTCP values over the whole cohort of patients when selecting the plan with the highest UTCP from the 2000 gMCO Pareto-optimal plans for each α value. The NTCP parameters for OARs are kept constant by using values in Table 2.

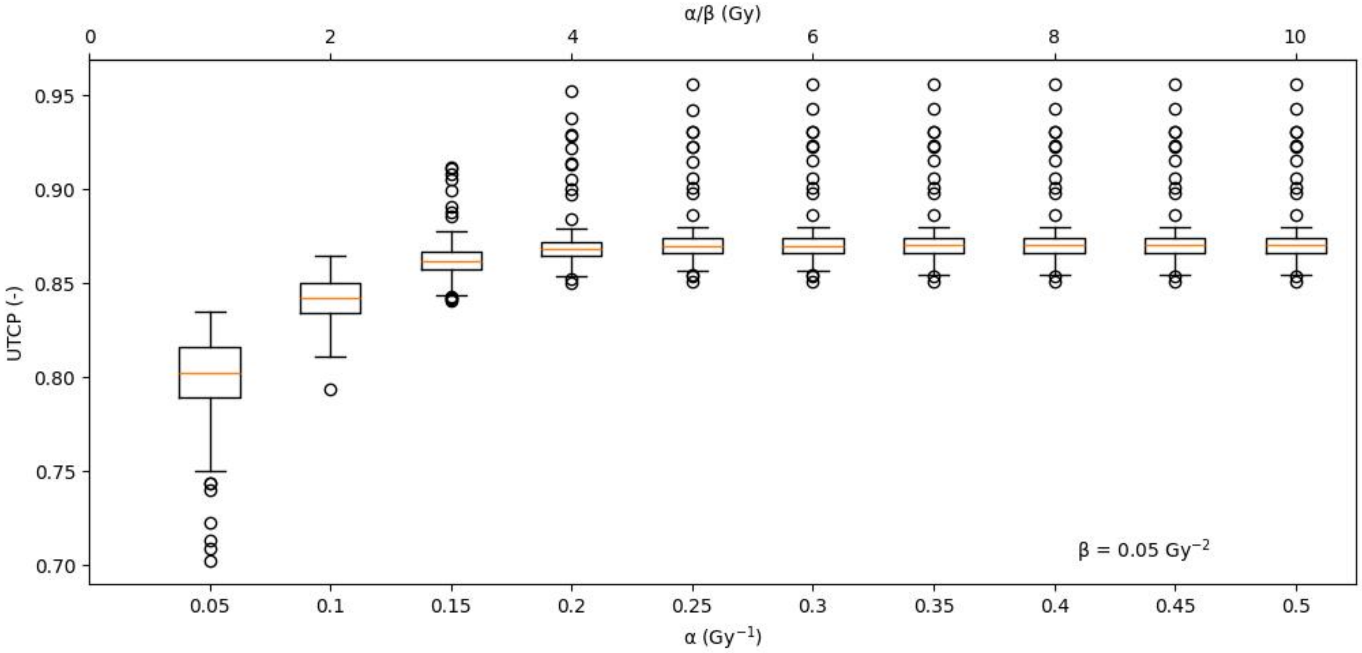

Figure S5: Impact of the α parameter (TCP model) for a 15 Gy HDR brachytherapy boost prescriptions while keeping a constant value of $\beta = 0.05$ Gy^{-2}. The boxplot shows UTCP values over the whole cohort of patients when selecting the plan with the highest UTCP from the 2000 gMCO Pareto-optimal plans for each α value. The NTCP parameters for OARs are kept constant by using values in Table 2.

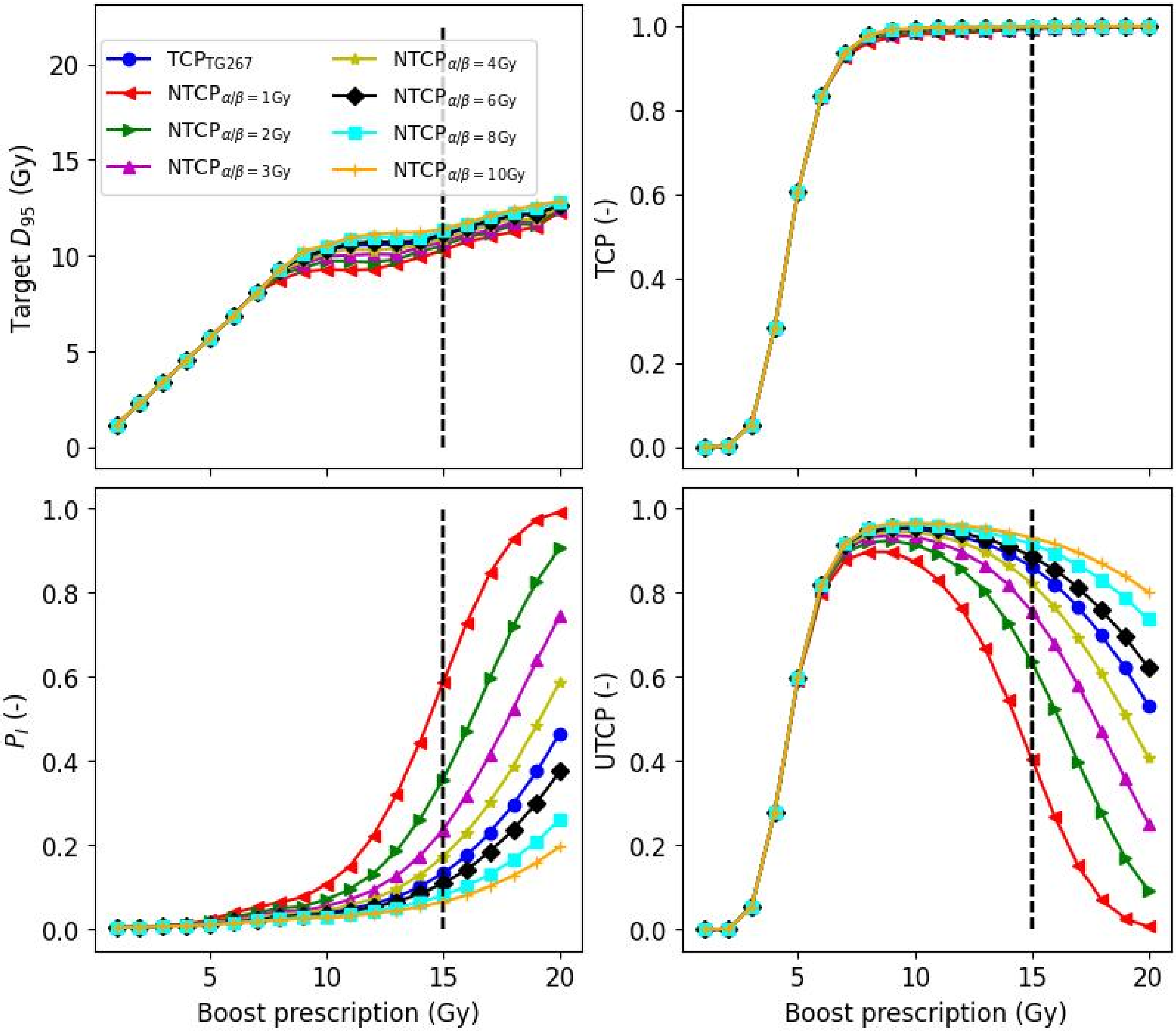

Figure S6: Impact of the radiobiological parameters (NTCP model) for different HDR brachytherapy boost prescriptions (delivered in a single fraction) to external beam radiation therapy (EBRT) on the (a) Target D_{95}, (b) TCP, (c) P_I, and (d) UTCP. The curves show median values over the whole cohort of patients when selecting plan with the highest UTCP from the 2000 gMCO Pareto-optimal plans for each prescription. The labels are defined by the value of α/β for OARs given constant TG-137 parameters for TCP. The black dashed lines illustrate the current boost prescription dose used at CHUQ-UL center (15 Gy).

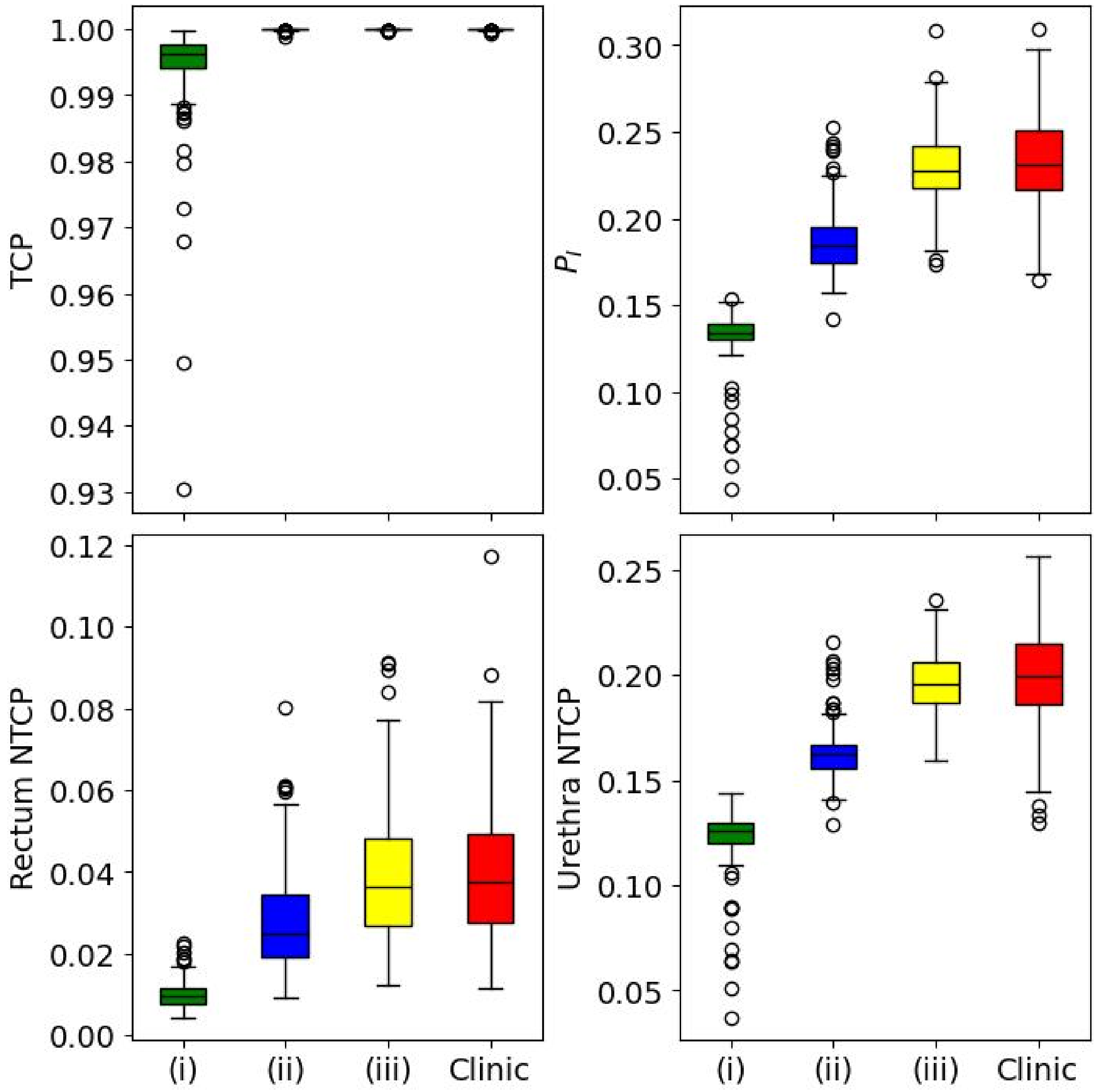

Figure S7: Companion of Figure 4. Radiobiological indices obtained with three different plan selection scenarios from 2000 gMCO Pareto-optimal plans with 15 Gy prescription dose (single fraction) as a boost to EBRT. Scenario (i) The UTCP is maximized without any physical dose constraints (green color). Scenario (ii) The UTCP is maximized while meeting criteria in Table 3 (blue color). Scenario (iii) The target coverage is maximized while meeting criteria in Table 3 (yellow color). The results of the clinical plans are also depicted (red color). Although not shown, bladder NTCP values were below 0.05 for all distributions.
